# Herb-partitioned moxibustion upregulated the expression of colonic epithelial tight junction-related proteins in Crohn’s disease model rats

**DOI:** 10.1186/s13020-016-0090-0

**Published:** 2016-04-26

**Authors:** Rong Ji, Anqi Wang, Haixia Shang, Liu Chen, Chunhui Bao, Luyi Wu, Huangan Wu, Yin Shi

**Affiliations:** College of Acumox and Tuina, Shanghai University of Traditional Chinese Medicine, Shanghai, 201203 China; Shanghai Research Institute of Acupuncture-Moxibustion and Meridian, Shanghai, 200030 China

## Abstract

**Background:**

Herb-partitioned moxibustion (HPM) at *Tianshu* (ST25) and *Qihai* (RN6) has been used to treat Crohn’s disease (CD). Injury to intestinal epithelial tight junctions (TJs) is the leading cause of CD onset with under expression of TJ-related proteins such as occludin, claudin-1, and zonula occludens protein-1 (ZO-1). This study aimed to investigate whether HPM can change the permeability of the intestinal epithelial barrier by affecting the expression of colonic epithelial TJ-related proteins in vitro.

**Methods:**

Forty-eight male Sprague–Dawley rats were randomly divided into four groups of twelve rats: normal control (NC) group; model control (MC) group; herb-partitioned moxibustion (HPM) group; and mesalazine control (MESA) group. The rats in the latter three groups were given trinitrobenzene sulfonic acid (TNBS) enemas to establish CD models. The HPM group was treated with HPM at *Tianshu* (ST25) and *Qihai* (RN6) once daily for 14 consecutive days, while the MESA group was given mesalazine solution (at the proportion of 0.018:1) by lavage twice daily for the same period. After the treatment period, the colon tissues from all groups were partly processed for macroscopic damage assessment and histological observation, and partly purified and cultured in vitro to examine the permeability of the intestinal epithelial cell barrier by trans-epithelial electrical resistance (TEER). Western blot and fluorescence quantitative polymerase chain reaction (FQ-PCR) analyses were performed to observe the expression of occludin, claudin-1, and ZO-1 proteins and mRNAs, respectively.

**Results:**

In the HPM and MESA groups, the typical CD macroscopic damage, i.e., inflammatory cell infiltration in colonic mucosa and submucosa, submucosal lymphoid follicular hyperplasia, hyperemia and edema, and morphological changes were improved to different degrees in the colonic tissues (HPM, MESA vs. MC for macroscopic score of colonic damage: all *P* < 0.001). The decreasing tendencies were minor for colonic TEER values (HPM, MESA vs. MC: all *P* < 0.001), and expression of intestinal epithelial TJ-related proteins (HPM, MESA vs. MC: all *P* < 0.05) and mRNAs (HPM, MESA vs. MC: all *P* < 0.05), especially in the HPM group (HPM vs. MESA for TEER values: *P* < 0.001).

**Conclusions:**

HPM at *Tianshu* (ST25) and *Qihai* (RN6) upregulated the expression of occludin, claudin-1, and ZO-1 in TNBS-induced CD model rats.

**Electronic supplementary material:**

The online version of this article (doi:10.1186/s13020-016-0090-0) contains supplementary material, which is available to authorized users.

## Background

Crohn’s disease (CD) is a chronic inflammatory bowel disease (IBD) characterized by intestinal inflammatory changes induced by abnormal immune responses of the host against the intestinal tolerated commensal microflora [1, 2]. Injury to the intestinal epithelial barrier (e.g., excessive apoptosis of intestinal epithelial cells) and genetic defects in the intestinal epithelial tight junctions (TJs) (e.g., under expression of TJ-related proteins) were reported to be involved in the occurrence and development of CD [3]. Intestinal mucosal tumor necrosis factor-alpha (TNF-α) and tumor necrosis factor receptor1 (TNFR1) were overactive in patients with mild to moderate CD [4, 5]. Malformation and dysfunction of intestinal epithelial TJs were found in patients with IBDs [6, 7], and related to under expression or abnormal distribution of intestinal epithelial TJ-related proteins [8].

Intestinal epithelial TJs are related to the permeability of the intestinal epithelial barrier [9, 10]. Changes in the permeability of the intestinal epithelial barrier are the leading pathologic alterations in CD [11, 12]. Occludin, claudin-1, and zonula occludens protein-1 (ZO-1) are major proteins for maintaining TJ permeability [8]. During active and non-active periods of CD, the expression of occludin, claudin-1, ZO-1 proteins and mRNAs were significantly reduced in the colonic mucosa, followed by increasing permeability of the intestinal epithelium and injury to the intestinal epithelial barrier [13, 14].

The goals in CD treatment are remission of inflammation, recovery of mucosa, prevention of relapse, avoidance of surgical intervention and minimization of the possibility of cancer development [15]. 5-Aminosalicylates (5-ASA) and thiopurines are clinically used in treatments for IBD [16]. 5-ASA preparations (e.g., mesalazine) are the first-line medicines for IBD treatment in mild to moderate ulcerative colitis (UC) [17]. However, they were found to be no more effective than placebo for active CD [18]. Therefore, the European Crohn’s and Colitis Organization has recognized the efficiency of mesalazine as “limited” [19]. Application of thiopurines in mild to moderate CD patients is limited because of their severe side effects including abnormities in liver and renal functions, digestive intolerance and withdrawal reactions [20]. In consideration of this, alternatives that offer safe and highly effective treatment are urgently needed for patients with mild to moderate CD.

Previous clinical and experimental studies showed that herb-partitioned moxibustion (HPM) was effective in relieving abdominal pain, diarrhea and other gastrointestinal symptoms, as well as improving the Crohn’s disease activity index in patients [4, 5]. HPM was able to significantly reduce the abnormal increases in TNF-α and TNFR1 in the intestinal mucosa [5]. HPM not only decreased the apoptosis of colonic epithelial cells, but also enhanced the expression of colonic epithelial TJ-related proteins in vitro [21, 22].

This study aims to investigate whether HPM would change the permeability of the intestinal epithelial barrier by affecting the expression of occludin, claudin-1,and ZO-1 in vitro.

## Methods

### Animals

Forty-eight male Sprague-Dawley rats (150 ± 10 g), aged 6–8 weeks, were supplied by the Experimental Animal Center, Shanghai University of Traditional Chinese Medicine (China). The experiments were approved by Ethics Committee of Shanghai University of Traditional Chinese Medicine (No. 2013025; Additional files 1 and 2). Each animal was free of any known pathogens and only used once. All rats were housed in a light-controlled room (12-h/12-h light/dark cycle with lights on at 07:00 am), with constant temperature (22 ± 1 °C) and humidity (42 ± 5 %). Prior to modeling, the rats were housed with six companions in each cage, and had free access to food and water. The animal studies were performed following the ARRIVE guideline (Additional file 3).

The 48 male rats were allocated into four groups by a randomized block design. After weighing on a scale, the rats were ranked according to their body weight, and the numbers 1 through 48 were written on the tails of the rats in ascending order. According to this order, sets off our rats with similar approximate weights comprised one block, and were simultaneously given 48 random numbers taken from the random number table (from line 6 row 8, left to right). The four rats in each block were rearranged by their random numbers into ascending order, and then allocated into four groups of twelve rats: normal control (NC) group; model control (MC) group; herb-partitioned moxibustion (HPM) group; and mesalazine control (MESA) group.

The CD models were prepared according to Morris’ method [23]. Briefly, all rats were given only water for 24 h before modeling. After being weighed on the scale, the rats in all four groups were anesthetized with sodium pentobarbital (30 mg/kg 2 %; Sigma Chemical Co., USA) by intraperitoneal injection. The rats in the MC, HPM, and MESA groups were given trinitrobenzene sulfonic acid (TNBS; Sigma Chemical Co., USA) enemas(0.5 mL/100 g; formula: TNBS/50 % ethanol = 2:1), while those in the NC group were given enemas with the same dose of normal saline. The rats were fixed in the handstand position for 1 min while the rubber tubes containing the enema solution were retained in the gut cavity at 6–8 cm in depth to ensure an adequate drug action, and the tubes were then withdrawn. This procedure was repeated on days 7, 14, 21, and 28. When the CD modeling procedure was completed, two rats from each group were arbitrarily selected (by picking a number out of a box) to examine whether the modeling was sufficient by observing hematoxylin and eosinstained colonic tissues under a light microscope (Leica Microsystems GmbH, Germany).

### Herbal cake and moxi cone preparation

The herbal powder formula was as follows: *Fuzi* (radix *Aconiti carmichaeli*) (10 g; Sichuan, China), *Rougui* (*Cinnamomum cassia Presl*) (2 g; Guanxi, China), *Danshen* (radix *Salviae miltiorrhizae*) (3 g; Anhui, China), *Honghua* (*Carthamus tinctorius* L.) (3 g; Henan, China), and *Muxiang* (*Saussurea costus*) (2 g; Yunnan, China). Decoction pieces of these traditional Chinese medicines were mixed, ground into a fine powder, passed through a 150-mesh sieve three times, and stored in dry and dark conditions at room temperature (25 °C). For use, 2.5 g of herbal powder was fully mixed with 3 g of *Shaoxing* wine into a thick paste, and then shaped into herbal cakes (1 cm in diameter, 0.6 cm in height) in a herbal cake mold for the experimental rats.

Moxi cones (10 mg in weight, 0.6 cm in diameter, 0.6 cm in height) were made by hand using refined mugwort floss (Huatuo, Suzhou, China).

### Treatments

After excluding the rats arbitrarily selected from each group to examine whether the CD modeling was sufficient, the remaining 10 rats in each group were processed as described below.

All rats received the same fixation before treatment. The devices were adjusted until the rats felt comfortable and stopped struggling. When HPM was applied to the HPM group, the rats in the other three groups received the same fixation for the same duration.

The NC and MC groups received no treatment. For the HPM group, the fur of the lower abdomen was carefully shaved to expose the chosen acupoints, *Tianshu* (ST25) and *Qihai* (RN6). The moxa cones were ignited with an incense stick after placement on the herbal cake at *Tianshu* (ST25) and *Qihai* (RN6). Three moxa cones were used for each rat in a single treatment for 10 min. The rats received the same treatment once daily for 14 days. For the MESA group, mesalazine solution (Dr. FalkPharma, Germany) at the proportion of 0.018:1 was fed by lavage [24]. The rats received the same treatment twice daily for 14 days.

### Assessment of macroscopic colonic damage

After the treatment period, all rats in the different groups were ethically euthanized with sodium pentobarbital (30 mg/kg 2 %; Sigma Chemical Co., USA) by intraperitoneal injection. Colonic tissues (2–3 cm in length; 6–8 cm away from the anus) were taken and rinsed 5–10 times with sterile phosphate-buffered saline (PBS) containing antibiotics. Assessment of macroscopic colonic damage was conducted according to previously published criteria [25], as shown in Table 1. The severity of inflammation was assessed using the same criteria.

**Table 1:** Criterion for assessment of colonic damage

	Macroscopic score	
Adhesions	None	0
	Minimal	1
	Involving several bowel loops	2
Strictures	None	0
	mild	2
	Severe, proximal dilatation	3
Ulcers	None	0
	Linear ulceration <1 cm	1
	Two linear ulcers <1 cm	2
	More sites of ulceration or one large ulcer >1 cm	3
Wall thickness	Less than 1 mm	0
	1–3 mm	1
	More than 1 mm	2
Maximum score		10

### Histological observation

Colonic tissues (1 cm) were removed for histological observation. The tissues were washed in ice-cold normal saline, fixed in 10 % formalin, embedded in paraffin, cut into 4 µm thin sections, and adhered onto plain glass slides. The sections were then baked in an oven at 58 °C for 24 h for dewaxing, stained with hematoxylin and eosin, dehydrated in 95, 90, and 70 % ethanol, cleaned up in xylene, mounted in Permount or Histoclad, and observed at 200× magnification under an Olympus CX31 optical microscope (Olympus, Japan) at 2.80 V and 2.74 A. Images were obtained with a Canon A640 camera (Canon, Japan).

### Establishment of an in vitro intestinal epithelial barrier model

The remaining tissues were cut into pieces with ophthalmic scissors, and digested with 0.4 % type IV collagenase for 2 h in an incubator at 37 °C and 5 % CO_2_. The supernatants were centrifuged at 5000×*g* for 10 min. After the supernatants were discarded, the cell pellets were resuspended and seeded into culture dishes with Dulbecco’s modified Eagle’s medium (DMEM; Sigma-Aldrich Chemical Co., USA) containing 10 % fetal bovine serum (Sigma Chemical Co., USA) in an incubator at 37 °C and 5 % CO_2_. The culture medium was changed every other day. Colonic epithelial cells were purified, digested with trypsin, and resuspended in complete medium before counting. Cells (1 × 10^3^) were taken and placed in 48-well plates for adherence and observation. Wells with high proportions of epithelial cells were expanded in culture. The above operations were repeated until most of the epithelial cells were visible under a light microscope (Leica Microsystems GmbH, Germany). The culture was continued for 7 days [26] until a complete epithelial barrier was formed from normal colonic epithelial cells. TEER, western blot, and fluorescence quantitative PCR (FQ-PCR) analyses of colonic epithelial cells were performed in each group.

### Measurement of colonic TEER

Cultured cells in the different groups were washed once with PBS and digested with 0.25 % trypsin [27, 28]. After adding culture medium and terminating the digestion process, the cells were counted and adjusted to a density of 2.5 × 10^5^ cells/mL. Transwell chambers for measuring transmembrane resistance were placed in 24-wellplates. Complete medium (600 µL) was added to the lower chamber, while a cell suspension (200 µL) was added to the upper chamber and cultured for 24 h at 37 °C and 5 % CO_2_. The medium in the upper and lower chambers of the transwell chambers was replaced with 200 and 600 µL of medium, respectively. A small chamber with medium only was set as a negative control. The plates were measured in a Millicell ERS-2 Epithelial Volt-Ohm Meter (Millipore, USA) after vertical plug-in of an electrode into three slots on the chamber wall. The average of these three values was taken for statistical analysis. The room temperature was strictly controlled at 25 °C, because TEER values were reported to be highly sensitive to the environmental temperature [29].

### Detection of occludin, claudin-1, and ZO-1 expression in the rat colonic epithelial cells cultured in vitro

Cells (2 × 10^6^) were taken from each group, added to a celllysis buffer (100 µL) until full cleavage was achieved, and boiled (100 °C) for 5 min. The insoluble material was removed by centrifugation (5000×*g* for 5 min). The proteins in each sample (50 µL) were separated by polyacrylamide gel electrophoresis and transferred to nitrocellulose membranes. The membranes were sequentially blocked with 5 % nonfat milk in PBS for 2 h and 5 % bovine serum albumin in PBS at 4 °C overnight, and then incubated with primary antibodies for 90 min at room temperature. The primary antibodies used were rabbit polyclonal immunoglobulin (IgG) antibodies against occludin (Ab31721; Abcam, UK; 1:250 dilution), claudin-1 (Ab15098; Abcam, UK; 1:25 dilution), and ZO-1 (Sc-10804; Santa Cruz Biotechnology, USA; 1:100 dilution). Peroxidase-conjugated goat anti-rabbit IgG (A0208; Beyotime, China; 1:1000 dilution) and chemiluminescence fluids A and B (1:1) were incubated with the membranes for 1 h and 1 min, respectively. The reactive bands were detected by chemiluminescent reagents (170-5061; Bio-Rad, USA). A Tanon-5200imager (Tanon, China) was employed for image acquisition, and Image-pro plus 6.0 software (MediaCybernetics, USA) was used to analyze the gray values of the bands. All the grey values of the bands were normalized by the value for glyceraldehyde-3-phosphate dehydrogenase (GAPDH).

### FQ-PCR detection of occludin, claudin-1, and ZO-1 mRNA expression in the rat colonic epithelial cells cultured in vitro

Total cellular RNA was separated from the rat colonic epithelial cell samples using Trizol reagent (1596-026; Invitrogen, USA). Three micrograms of the total RNA wasused as a template for reverse transcription of cDNAs. The cDNAs were synthesized by reverse transcription using a RevertAid cDNA Synthesis Kit (#K1622; Fermentas, USA) in accordance with the manufacturer’s instructions, and then amplified, the primer sequences used for PCR amplification reaction were shown in Table 2. The following cycling conditions were used: reverse transcription at 37 °C for 1 h, inactivation of the MMLV RT enzyme at 95 °C for 5 min, and 40 cycles of 50 °C for 2 min, 95 °C for 5 min, 95 °C for 15 s, and 60 °C for 45 s. Real-time PCR was performed with a QuantiTect SYBR Green PCR Kit (#K0223; Thermo Fisher Scientific, USA) using an ABI 7500 real-time PCR system and SDS software (Applied Biosystems, USA). Data for the mRNA levels were calculated as the relative amounts normalized to that of GAPDH.

**Table 2:** The sequence of the forward primer and reverse primer used for FQ-PCR assays

Name	Primer sense	Primer sequence (5′ → 3′)	Amplification product (bp)
GAPDH	Forward	5′-CCGAGGGCCCACTAAAGG-3′	116
	Reverse	5′-GCTGTTGAAGTCACAGGAGACAA-3′	
Occludin	Forward	5′-GGGACAGAGCCTATGGAA-3′	191
	Reverse	5′-GGAAGCGATGAAGCAAAA-3′	
Claudin-1	Forward	5′-CTGGCTTCGCTGGGATGGA -3′	100
	Reverse	5′-TGGCCTGAGCAGTCACGATGTT-3′	
ZO-1	Forward	5′-GATGAGCGGGCTACCTTA-3′	126
	Reverse	5′-ATGGGAGCGAACTGAATG-3′	

### Statistical analysis

Statistical data were represented in graphs as mean ± standard deviation (SD). Statistical analyses were performed using SPSS 16.0 software (SPSS, USA). Differences within experimental groups were compared by one-way analysis of variance (ANOVA). LSD test was performed for multiple group comparison. Values of *P* *<* 0.05 were considered statistically significant.

## Results

### Colonic histopathological observations in the different groups

**Fig. 1:**
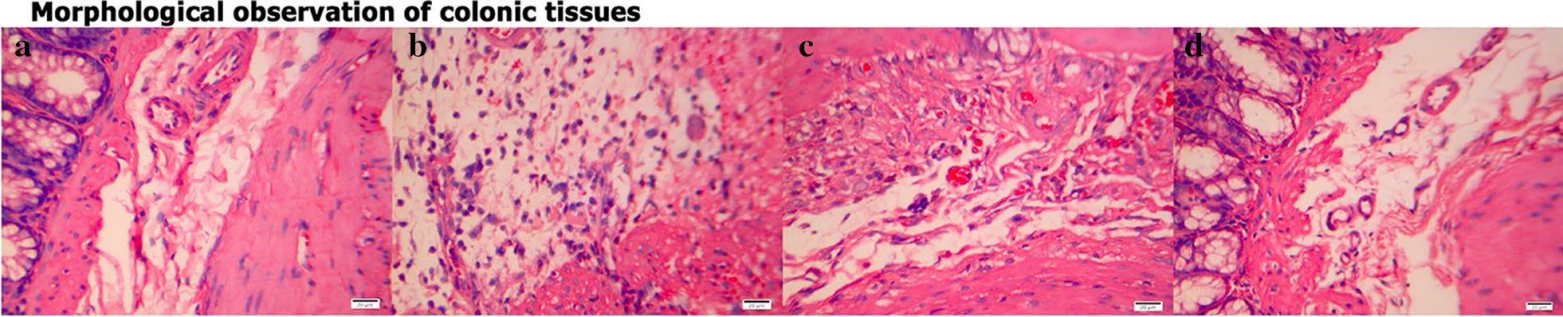
Histopathological observation of the colon tissue. Hematoxylin and eosin staining method, ×200. **a** NC group; **b** MC group; **c** MESA group; **d** HPM group

In the normal group (Fig. 1a), the colonic mucosa was intact, and the morphology of submucosal and muscular tissues was normal. In the MC group (Fig. 1b), there were large numbers of eosinophils with marked inflammatory cell infiltration in colonic mucosa and submucosa, and obvious submucosal lymphoid follicular hyperplasia, epithelial damage, submucosal hyperemia and edema, fibroblast proliferation, and muscle tissue damage, as well as epithelioid reactions, giant cells, and other typical pathological intestinal changes of CD. In the MESA group (Fig. 1c), there were eosinophils and inflammatory cell infiltration in colonic mucosa and submucosa, submucosal hyperemia and edema, fibroblast proliferation, small amounts of epithelioid reactions and giant cells, and slightly disordered layers of tissue morphology. In the HPM group (Fig. 1d), there were only small amounts of inflammatory cell infiltration in colonic mucosa and submucosa, mild hyperemia, small amounts of fibroblast proliferation, and normal morphology of mucosal, submucosal, and muscular tissues.

### Scores for macroscopic colonic damage in the different groups

**Fig. 2:**
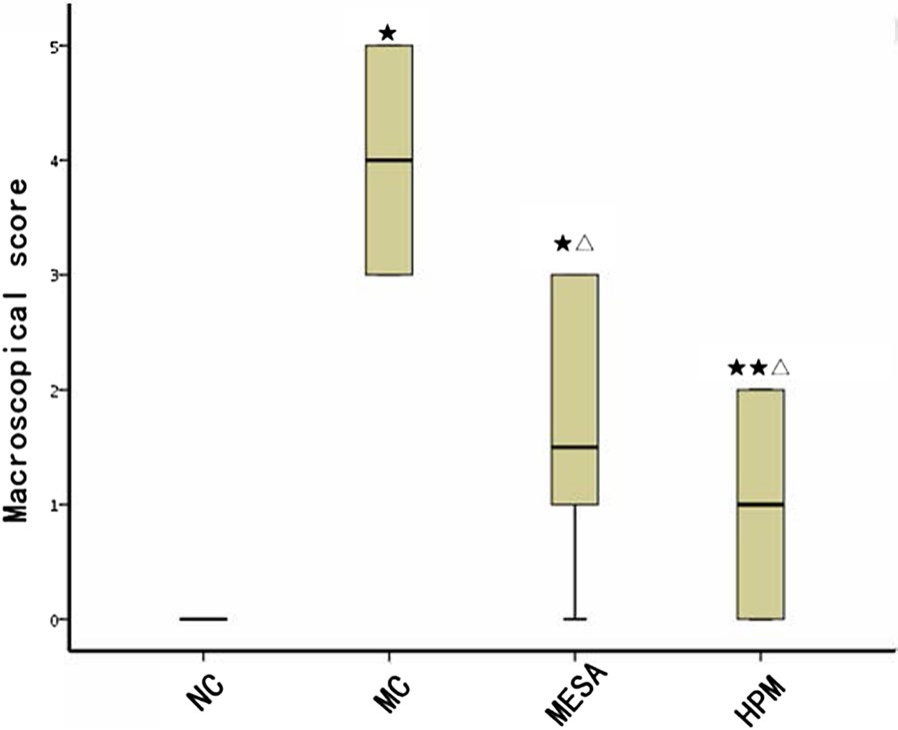
Macroscopic score of colonic damage. *NC* normal control group, *MC* model control group, *MESA* mesalazine group, and *HPM* herb-partitioned moxibustion group. ★*P* < 0.001 versus NC, ★★*P *< 0.05 versus NC; △*P* < 0.001 versus MC. The data of macroscopical score do not have normal distribution, the *box-plot* is used to express, as shown below. The NC group has the same value, so the *box-plot* only have one straight line. The maximum value is equal to the upper quantile and the minimum value is equal to the lower quantile in MC group. The maximum value is equal to the upper quantile in MESA group. The maximum value is equal to the upper quantile and the minimum value is equal to the lower quantile in HPM group

Compared with the NC group, the MC, MESA, and HPM groups showed significant increases in the macroscopic scores for colonic damage (*P* = 0.000, *P* = 0.000, *P* = 0.024; Fig. 2). However, the macroscopic scores for colonic damage in the MESA and HPM groups were significantly lower than that in the MC group (*P* = 0.000, *P* = 0.000), although there was no significant difference between these two groups (*P* = 0.075). Both HPM and MESA therapy could significantly improve the macroscopic colonic mucosal damage in CD.

### Comparison of TEER values in the different groups

**Fig. 3:**
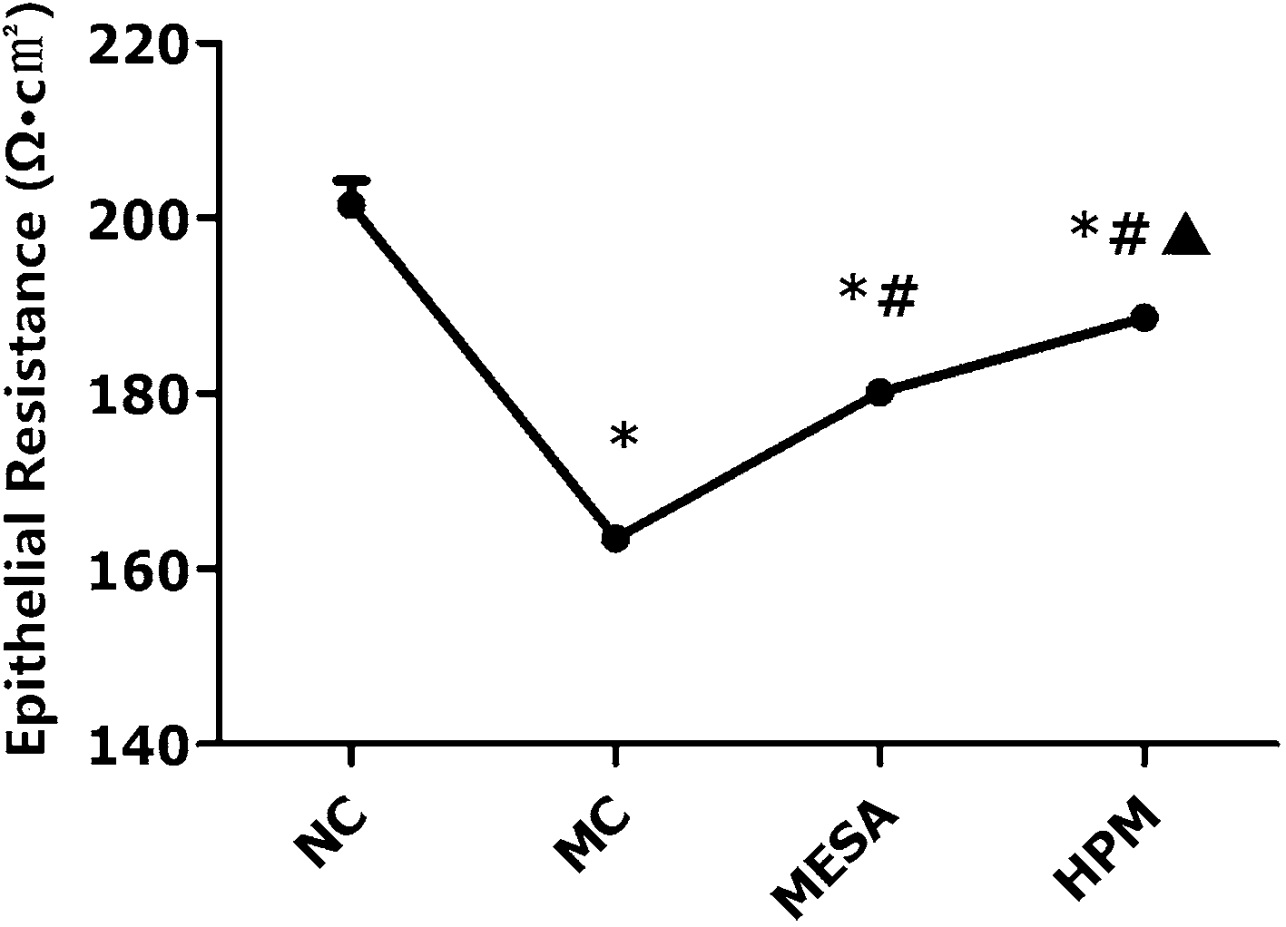
Transepithelial electrical resistance values of each group. *NC* normal control group, *MC* model control group, *MESA* mesalazine group and *HPM* herb-partitioned moxibustion group. ^∗^ *P* < 0.01 versus NC; ^#^ *P* < 0.01 versus MC; ^▲^ *P* < 0.01 versus MESA. The standard deviation in MC group (163.53 ± 0.77), MESA group (180.13 ± 1.09) and HPM group (188.60 ± 1.07) is small and not shown in the *graph*

As shown in Fig. 3, there was a significant decrease in the colonic TEER values in the MC group compared with the NC group (*P* = 0.000). There were significant increases in the colonic TEER values in both the HPM and MESA groups compared with the MC group (*P* = 0.000, *P* = 0.000), while the HPM group had significantly better TEER values than the MESA group (*P* = 0.000).There were also significant differences in the HPM and MESA groups compared with the NC group (*P* = 0.000, *P* = 0.000).

### Expression of occludin, claudin-1, and ZO-1 proteins in the different groups

**Fig. 4:**
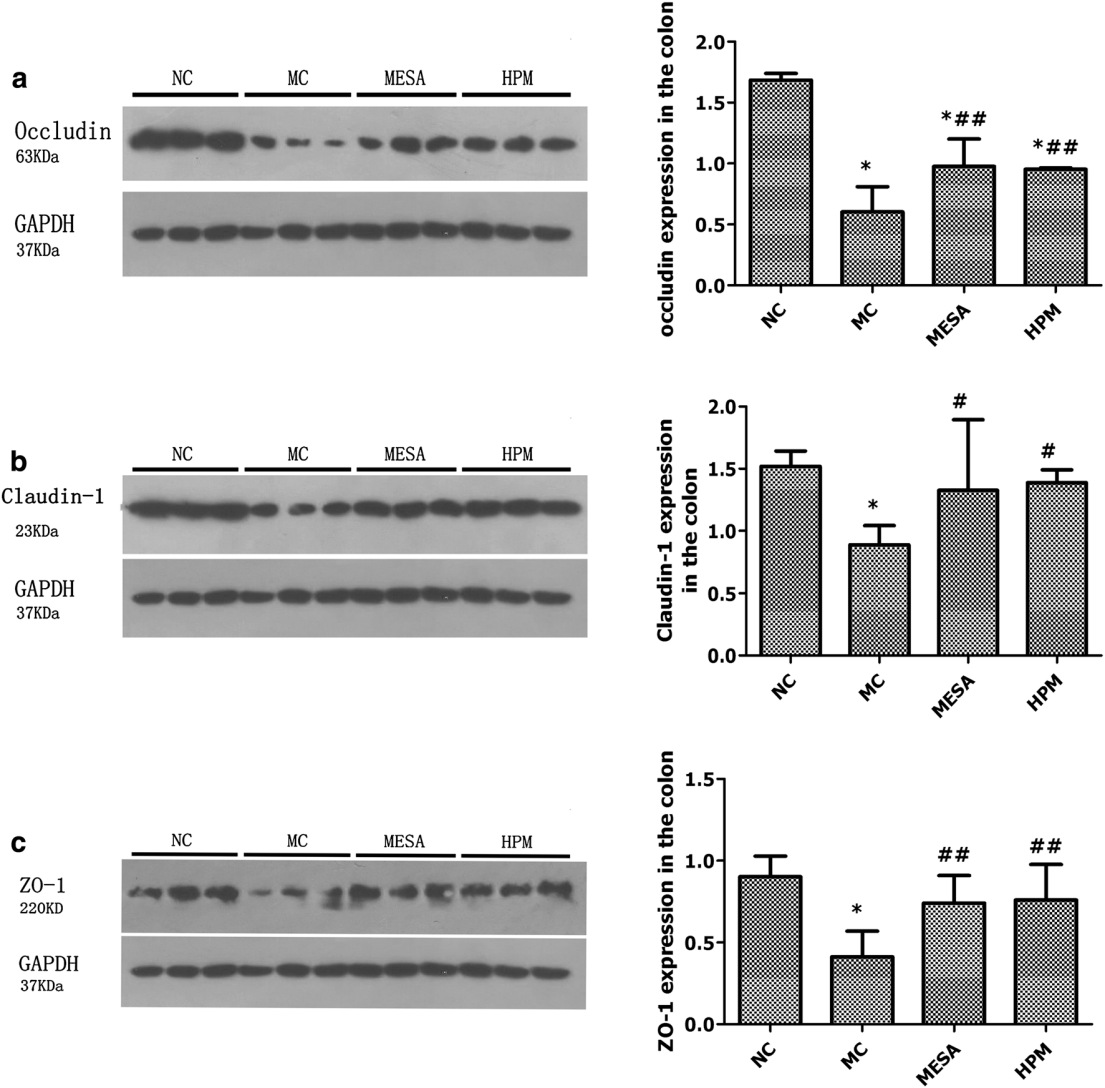
Expression of colonic epithelial TJ-related proteins occludin, claudin-1 and ZO-1 in each group. **a** Expression of occludin in each group. **b** Expression of claudin-1 in each group. **c** Expression of ZO-1 in each group. *NC* normal control group, *MC* model control group, *MESA* mesalazine group, and *HPM* herb-partitioned moxibustion group. ^∗^ *P* < 0.01 versus NC; ^#^ *P* < 0.01 versus MC; ^##^ *P* < 0.05 versus MC

For occludin protein expression (Fig. 4a), there was a significant decrease in the MC group compared with the NC group (*P* = 0.000), significant increases in the HPM and MESA groups compared with the MC group (*P* = 0.025, *P* = 0.019), and no significant difference between the HPM group and the MESA group (*P* = 0.859), but significant differences in the HPM and MESA groups compared with the NC group (*P* = 0.001, *P* = 0.000). For claudin-1 protein expression (Fig. 4b), there was a significant decrease in the MC group compared with the NC group (*P* = 0.000), significant increases in the HPM and MESA groups compared with the MC group (*P* = 0.001, *P* = 0.002), no significant difference between the HPM group and the MESA group (*P* = 0.535), and no significant differences in the HPM and MESA groups compared with the NC group (*P* = 0.196, *P* = 0.073). For ZO-1 protein expression (Fig. 4c), there was a significant decrease in the MC group compared with the NC group (*P* = 0.008), significant increases in the HPM and MESA groups compared with the MC group (*P* = 0.038, *P* = 0.046), no significant difference between the HPM group and the MESA group (*P* = 0.899), and no significant differences in the HPM and MESA groups compared with the NC group (*P* = 0.336, *P* = 0.282).

### Expression of occludin, claudin-1, and ZO-1 mRNAs in the different groups

**Fig. 5:**
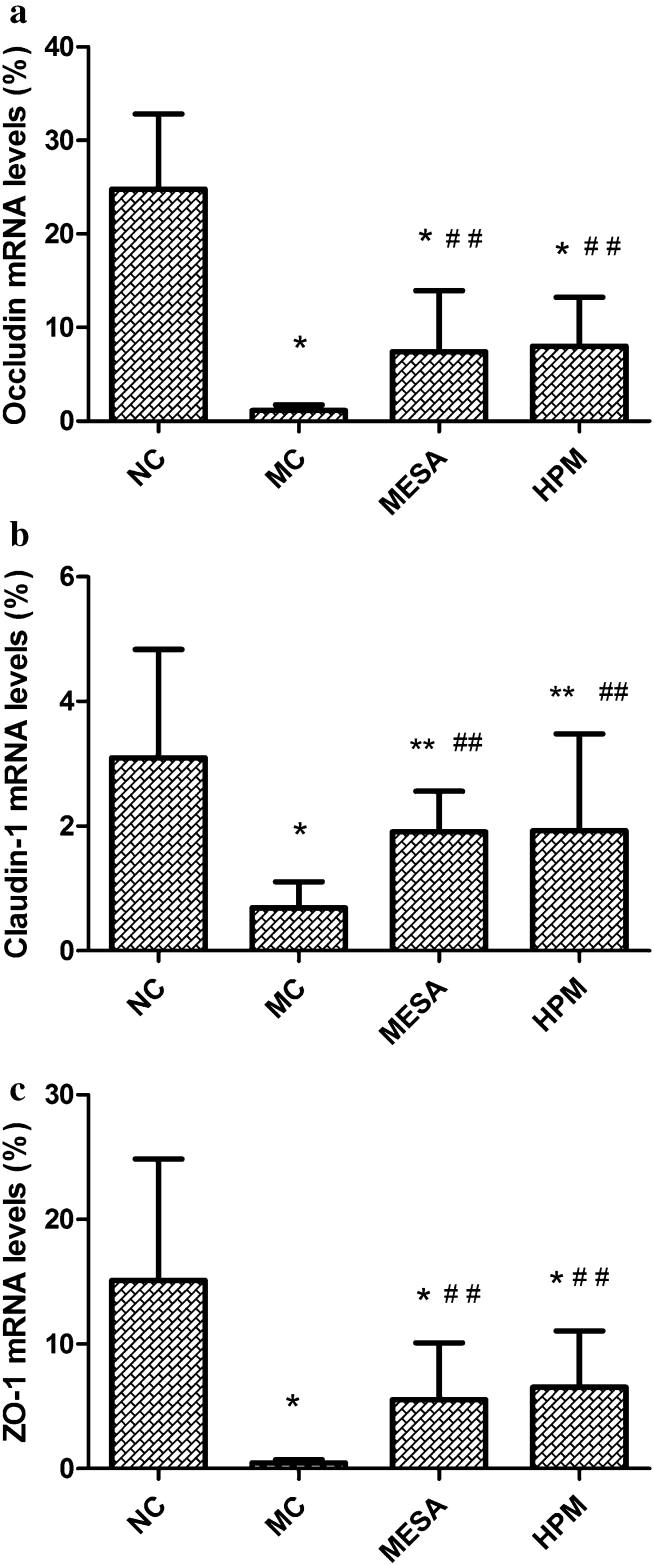
mRNA levels of colonic epithelial TJ-related protein occludin, claudin-1 and ZO-1 in each group. **a** mRNA levels of occludin in each group. **b** mRNA levels of claudin-1 in each group. **c** mRNA levels of ZO-1 in each group.* NC* normal control group, *MC* model control group, *MESA* mesalazine group, and *HPM* herb-partitioned moxibustion group. ^∗^ *P* < 0.01 versus NC; ^∗∗^ *P* < 0.05 versus NC; ^##^ *P* < 0.05 versus MC

For occludin mRNA expression (Fig. 5a), there was a significant decrease in the MC group compared with the NC group (*P* = 0.000), significant increases in the HPM and MESA groups compared with the MC group (*P* = 0.028, *P* = 0.043), and no significant difference between the HPM group and the MESA group (*P* = 0.846), but still significant differences in the HPM and MESA groups compared with the NC group (*P* = 0.000, *P* = 0.000). For claudin-1 mRNA expression (Fig. 5b), there was a significant decrease in the MC group compared with the NC group (*P* = 0.000), significant increases in the HPM and MESA groups compared with the MC group (*P* = 0.035, *P* = 0.039), and no significant difference between the HPM group and the MESA group (*P* = 0.968), but still significant differences in the HPM and MESA groups compared with the NC group (*P* = 0.048, *P* = 0.044). For ZO-1 mRNA expression (Fig. 5c), there was a significant decrease in the MC group compared with the NC group (*P* = 0.000), significant increases in the HPM and MESA groups compared with the MC group (*P* = 0.017, *P* = 0.044), and no significant difference between the HPM group and the MESA group (*P* = 0.684), but still significant differences in the HPM and MESA groups compared with the NC group (*P* = 0.001, *P* = 0.000).

## Discussion

This study has demonstrated the effects of HPM on repairing the colonic epithelial barrier and upregulating the expression of occludin, claudin-1, and ZO-1 in vitro. In the study, we first observed both macroscopic and histological damage to the colonic tissues from the different groups. Immediately after the colonic tissues were removed and washed, we assessed the scores for adhesions, strictures, ulcers, and wall thickness of the colonic tissues. In the NC group, the colonic tissues showed mild hyperemia and edema, but no mucosal erosion or ulcers, which might have arisen through stimulation during the surgery process. Almost no signs of tissue adhesions and strictures were observed, and the wall thickness was normal. In the MC group, severe hyperemia and edema were generally observed in most rats, while deep and multiple ulcers were common. In several rats, we also observed slight adhesions of the adjacent colonic tissues, accompanied by loss of elasticity and smoothness of colonic tissues, and slight increases in wall thickness. This was because that our CD modeling procedure could only imitate the incipient acute colonic inflammation, which is characterized by mucosal hyperemia, edema, and ulcers. However, the symptoms of tissue adhesions, strictures, and intestinal wall stiffness were mainly the results of chronic recurrent inflammation. Compared with the MC group, the MESA and HPM groups showed remarkable signs of inflammation remission, as the mucosal hyperemia, edema, and ulcers were moderate and localized, and adhesions and strictures were barely observed.

By light microscopy, we observed pathological changes of the colonic tissues in the CD, HPM, and MESA groups. In tissues taken from the MC group, eosinophils and inflammatory cell infiltration in colonic mucosa and submucosa, and obvious submucosal hyperemia and edema, as well as obvious lymphoid follicles, fibroblast proliferation, and other typical CD pathological changes were observed under a light microscope. After treatment with mesalazine, these pathological changes were not improved. After treatment with HPM, there was only a small amount of inflammatory cell infiltration in colonic mucosa and submucosa, mild hyperemia, and scattered fibroblast proliferation among normal morphology of the mucosal, submucosal, and muscular tissues.

We then examined the TEER of the in vitro cultured colonic epithelial cells from the different groups. TEER determination is widely used to functionally analyze TJ dynamics in cell culture models of physiological barriers [30–32], which can be used to evaluate the paracellular permeability of epithelial monolayers. A decrease in TEER was associated with disruption of TJs and subtle changes in the cytoskeletal structure, resulting in a subsequent increase in the paracellular permeability of the epithelial monolayer [33]. In the present study, we found significant decreases in the TEER values in the MC group, but increases to different degrees in the HPM and MESA groups, with the HPM group showing larger rising trends. These findings suggested that during the pathological process of CD, the structure of TJs was impaired, leading to a subsequent decrease in the paracellular permeability of the epithelial monolayer. However, both HPM and mesalazine treatment can recover or reduce the abnormally increased colonic epithelial permeability in CD, and HPM possesses the advantages of repairing TJs and increasing the permeability of the epithelial monolayer.

The intestinal epithelium creates a chemical and mechanical barrier that separates the host from microbes [34]. The chemical barrier consists of mucus, peptides, and antimicrobial proteins secreted by goblet cells and Paneth cells, while the mechanical barrier is composed of different kinds of enterocytes and TJs to join the cells together. These barriers are responsible for permeability, i.e., selective absorption of water and nutrients in the gut lumen through either transepithelial or paracellular transport. TJs are multimolecular complexes that seal the intercellular space between adjacent epithelial cells [35] and act as a diffusion barrier to control the transportation of ions, macromolecules, and immune cells in the paracellular pathway [36], and are therefore the major determinants of epithelial permeability [37]. TJs maintain symbiosis within intestinal microflora and prevent infection or inflammation induced by bacterial translocation [38].

The TJ-related proteins of the intestinal epithelial barrier are mainly located at the top side of the enteric cavity among intestinal epithelial cells, then zonally surround the side wall and seal up the intestinal epithelial gap. TJs are composed of three transmembrane protein families [39]: claudins, junctional adhesion molecule (JAM) proteins, and TJ-associated Marvel domain proteins (TAMPs) including occludin, tricellulin, and Marvel D3. Claudins control the charge and size-selectivity of TJs, while JAMs and TAMPs stabilize TJs and maintain epithelial permeability. ZO-1 connects the multiprotein structure of TJs to peri-junctional actomyosin. These three protein families have overlapping and unique functions in the regulation of TJs [40]. In this study, occludin, claudin-1, and ZO-1 were formed into complex proteins by binding between the C-terminals of the TJ transmembrane proteins claudin-1 and occludin and the N-terminals of the cytoplasmic-attached protein ZO-1 [41], thus allowing their external parts to interact with the transmembrane junction proteins of adjacent cells. At the same time, the C-terminals of ZO-1 were connected with actin of the cytoplasmic cytoskeleton, making it possible for claudin-1 to act on these actin molecules, and thus participate in maintaining the stability and selective permeability of TJs [42]. Occludin is considered to be fundamental to the structure of TJs [43]. Knockout of the occludin gene [28] in epithelial cells can cause similar pathological intestinal changes to IBD in animals. Reduced expression of intestinal occludin protein and mRNA was observed in patients with ulcerative colitis and CD [3], suggesting the possible involvement of occludin in the pathogenesis of IBD. In the present study, in vitro experiments demonstrated radical decreases in colonic epithelial occludin protein and mRNA expression in the NC group, compared with remarkable recovery of the occludin protein and mRNA expression in the MESA and HPM groups. The same tendency was observed for the other two TJ-related proteins, claudin-1 and ZO-1.

## Conclusions

HPM at *Tianshu* (ST25) and *Qihai* (RN6) upregulated the expression of occludin, claudin-1, and ZO-1 in TNBS-induced CD model rats.

## Additional files

10.1186/s13020-016-0090-0 Ethical approval document in Chinese.
10.1186/s13020-016-0090-0 Ethical approval documents in English.
10.1186/s13020-016-0090-0 The ARRIVE guidelines.

## Abbreviations

CD: Crohn’s disease
IBD: inflammatory bowel disease
TJ: tight junction
TNF-α: tumor necrosis factor-alpha
TNFR1: tumor necrosis factor receptor-1
ZO-1: zonula occludens proteins-1
UC: ulcerative colitis
ECCO: European Crohn’s and Colitis Organization
SD: Sprague–Dawley
TNBS: trinitrobenzene sulfonic acid
NC: normal control
MC: model control
HPM: herb-partitioned moxibustion
MESA: mesalazine
FQ-PCR: fluorescent quantitative polymerase chain reaction
DMEM: Dulbecco’s modified Eagle’s medium
PBS: phosphate buffer saline
RT: room temperature
GAPDH: glyceraldehyde-3-phosphate dehydrogenase
SD: standard deviation
ANOVA: one way analysis of variance
JAM: junctional adhesion molecule
TAMPs: TJ-associated Marvel domain proteins

## References

[1] Kucharzik T, Lugering N, Rautenberg K, Lugering A, Schmidt MA, Stoll R, Domschke W (2000). Role of M cells in intestinal barrier function. Ann N Y Acad Sci.

[2] Cosnes J, Beaugerie L, Carbonnel F, Gendre JP (2001). Smoking cessation and the course of Crohn’s disease: an intervention study. Gastroenterology.

[3] Wu HG, Zhou LB, Shi DR, Liu SM, Liu HR, Zhang BM, Chen HP, Zhang LS (2000). Morphological study on colonic pathology in ulcerative colitis treated by moxibustion. World J Gastroenterol.

[4] Shi Y, Wu HG (2003). The clinical study on herbs-partitioned moxibustion treatment of Crohn’s disease. JiangXi J TCM.

[5] Shi Y, Bao CH, Wu HG, Chen WF, Qin XD, Zhang R, Wu LY (2011). Effect of herbs-partitioned moxibustion combined with acupuncture on the expressions of intestinal mucosa TNF-α, TNFR1, TNFR2 and apoptosis of intestinal epithelial cells in Crohn’s disease patients. Shanghai J TCM.

[6] Amasheh M, Grotjohann I, Amasheh S, Fromm A, Soderholm JD, Zeitz M, Fromm M, Schulzke JD (2009). Regulation of mucosal structure and barrier function in rat colon exposed to tumor necrosis factor alpha and interferon gamma in vitro: a novel model for studying the pathomechanisms of inflammatory bowel disease cytokines. Scand J Gastroenterol.

[7] Gassler N, Rohr C, Schneider A, Kartenbeck J, Bach A, Obermuller N, Otto HF, Autschbach F (2001). Inflammatory bowel disease is associated with changes of enterocytic junctions. Am J Physiol Gastrointest Liver Physiol.

[8] Poritz LS, Garver KI, Tilberg AF, Koltun WA (2004). Tumor necrosis factor alpha disrupts tight junction assembly. J Surg Res.

[9] McGuckin MA, Eri R, Simms LA, Florin TH, Radford-Smith G (2009). Intestinal barrier dysfunction in inflammatory bowel diseases. Inflamm Bowel Dis.

[10] Edelblum KL, Turner JR (2009). The tight junction in inflammatory disease: communication breakdown. Curr Opin Pharmacol.

[11] May GR, Sutherland LR, Meddings JB (1993). Is small intestinal permeability really increased in relatives of patients with Crohn’s disease?. Gastroenterology.

[12] Schmitz H, Barmeyer C, Fromm M, Runkel N, Foss HD, Bentzel CJ, Riecken EO, Schulzke JD (1999). Altered tight junction structure contributes to the impaired epithelial barrier function in ulcerative colitis. Gastroenterology.

[13] Oshitani N, Watanabe K, Nakamura S, Fujiwara Y, Higuchi K, Arakawa T (2005). Dislocation of tight junction proteins without F-actin disruption in inactive Crohn’s disease. Int J Mol Med.

[14] Kucharzik T, Walsh SV, Chen J, Parkos CA, Nusrat A (2001). Neutrophil transmigration in inflammatory bowel disease is associated with differential expression of epithelial intercellular junction proteins. Am J Pathol.

[15] Gisbert JP, Chaparro M, Gomollon F (2011). Common misconceptions about 5-aminosalicylates and thiopurines in inflammatory bowel disease. World J Gastroenterol.

[16] Iacucci M, Ghosh S (2010). Mesalazine in inflammatory bowel disease: a trendy topic once again. Canadian J Gastroenterol.

[17] Sutherland L, Macdonald JK. Oral 5-aminosalicylic acid for induction of remission in ulcerative colitis. The Cochrane database of systematic reviews. 2006: CD000543. 10.1002/14651858.CD000543.pub2 16625536

[18] Feagan BG (2004). 5-ASA therapy for active Crohn’s disease: old friends, old data, and a new conclusion. Clin Gastroenterol Hepatol.

[19] Dignass A, Van Assche G, Lindsay JO, Lemann M, Soderholm J, Colombel JF, Danese S, D’Hoore A, Gassull M, Gomollon F (2010). The second European evidence-based consensus on the diagnosis and management of Crohn’s disease: current management. J Crohn’s Colitis.

[20] Terdiman JP, Gruss CB, Heidelbaugh JJ, Sultan S, Falck-Ytter YT (2013). Practice AGAIC, Quality Management C: american Gastroenterological Association Institute guideline on the use of thiopurines, methotrexate, and anti-TNF-alpha biologic drugs for the induction and maintenance of remission in inflammatory Crohn’s disease. Gastroenterology.

[21] Bao CH, Wu LY, Shi Y, Wu HG, Liu HR, Zhang R, Yu LQ, Wang JH (2011). Moxibustion down-regulates colonic epithelial cell apoptosis and repairs tight junctions in rats with Crohn’s disease. World J Gastroenterol.

[22] Shi Y, Bao CH, Wu HG, Ma XP, Yu LQ, Zhang R, Chen WF (2011). Effect of moxibustion on colonic TNF-alpha content and influence of colonic supernatant of crohn’s disease rats undergoing moxibustion on expression of occludin, claudin-1 and zonula occludens-1 proteins and genes in cultured colonic epithelial cells. Acupuncture Res.

[23] Morris GP, Beck PL, Herridge MS, Depew WT, Szewczuk MR, Wallace JL (1989). Hapten-induced model of chronic inflammation and ulceration in the rat colon. Gastroenterology.

[24] Xu SY, Bian RL, Chen X: Experimental Methodology of Pharmacology. Beijing: People’s Medical Publishing House; 1982 .p. 1184.

[25] Vilaseca J, Salas A, Guarner F, Rodriguez R, Martinez M, Malagelada JR (1990). Dietary fish oil reduces progression of chronic inflammatory lesions in a rat model of granulomatous colitis. Gut.

[26] Yeh KY, Chopra DP (1980). Epithelial cell cultures from the colon of the suckling rat. In Vitro.

[27] Ma TY, Nguyen D, Bui V, Nguyen H, Hoa N (1999). Ethanol modulation of intestinal epithelial tight junction barrier. Am J Physiol.

[28] Schmitz H, Fromm M, Bentzel CJ, Scholz P, Detjen K, Mankertz J, Bode H, Epple HJ, Riecken EO, Schulzke JD (1999). Tumor necrosis factor-alpha (TNFalpha) regulates the epithelial barrier in the human intestinal cell line HT-29/B6. J Cell Sci.

[29] Blume LF, Denker M, Gieseler F, Kunze T (2010). Temperature corrected transepithelial electrical resistance (TEER) measurement to quantify rapid changes in paracellular permeability. Pharmazie.

[30] Muendoerfer M, Schaefer UF, Koenig P, Walk JS, Loos P, Balbach S, Eichinger T, Lehr CM (2010). Online monitoring of transepithelial electrical resistance (TEER) in an apparatus for combined dissolution and permeation testing. Int J Pharm.

[31] Balda MS, Whitney JA, Flores C, Gonzalez S, Cereijido M, Matter K (1996). Functional dissociation of paracellular permeability and transepithelial electrical resistance and disruption of the apical-basolateralintramembrane diffusion barrier by expression of a mutant tight junction membrane protein. J Cell Biol.

[32] Klingberg TD, Pedersen MH, Cencic A, Budde BB (2005). Application of measurements of transepithelial electrical resistance of intestinal epithelial cell monolayers to evaluate probiotic activity. Appl Environ Microbiol.

[33] Narai A, Arai S, Shimizu M (1997). Rapid decrease in transepithelial electrical resistance of human intestinal Caco-2 cell monolayers by cytotoxic membrane perturbents. Toxicol In Vitro.

[34] Stappenbeck TS, Rioux JD, Mizoguchi A, Saitoh T, Huett A, Darfeuille-Michaud A, Wileman T, Mizushima N, Carding S, Akira S (2011). Crohn disease: a current perspective on genetics, autophagy and immunity. Autophagy.

[35] Weber CR, Turner JR (2007). Inflammatory bowel disease: is it really just another break in the wall?. Gut.

[36] Furuse M (2010). Molecular basis of the core structure of tight junctions. Cold Spring Harb Perspect Biol.

[37] Gunzel D, Yu AS (2013). Claudins and the modulation of tight junction permeability. Physiol Rev.

[38] Farhadi A, Banan A, Fields J, Keshavarzian A (2003). Intestinal barrier: an interface between health and disease. J Gastroenterol Hepatol.

[39] Rodgers LS, Beam MT, Anderson JM, Fanning AS (2013). Epithelial barrier assembly requires coordinated activity of multiple domains of the tight junction protein ZO-1. J Cell Sci.

[40] Shen L (2012). Tight junctions on the move: molecular mechanisms for epithelial barrier regulation. Ann N Y Acad Sci.

[41] Hermiston ML, Gordon JI (1995). Inflammatory bowel disease and adenomas in mice expressing a dominant negative N-cadherin. Science.

[42] Gonzalez JE, DiGeronimo RJ, Arthur DE, King JM (2009). Remodeling of the tight junction during recovery from exposure to hydrogen peroxide in kidney epithelial cells. Free Radic Biol Med.

[43] Blasig IE, Bellmann C, Cording J, Del Vecchio G, Zwanziger D, Huber O, Haseloff RF (2011). Occludin protein family: oxidative stress and reducing conditions. Antioxid Redox Signal.

