# Supplementary figures and images for: Herb-partitioned moxibustion upregulated the expression of colonic epithelial tight junction-related proteins in Crohn’s disease model rats
Source: Chin Med. 2016 Apr 26;11:20. doi: 10.1186/s13020-016-0090-0 (PMC4845475; doi:10.1186/s13020-016-0090-0)

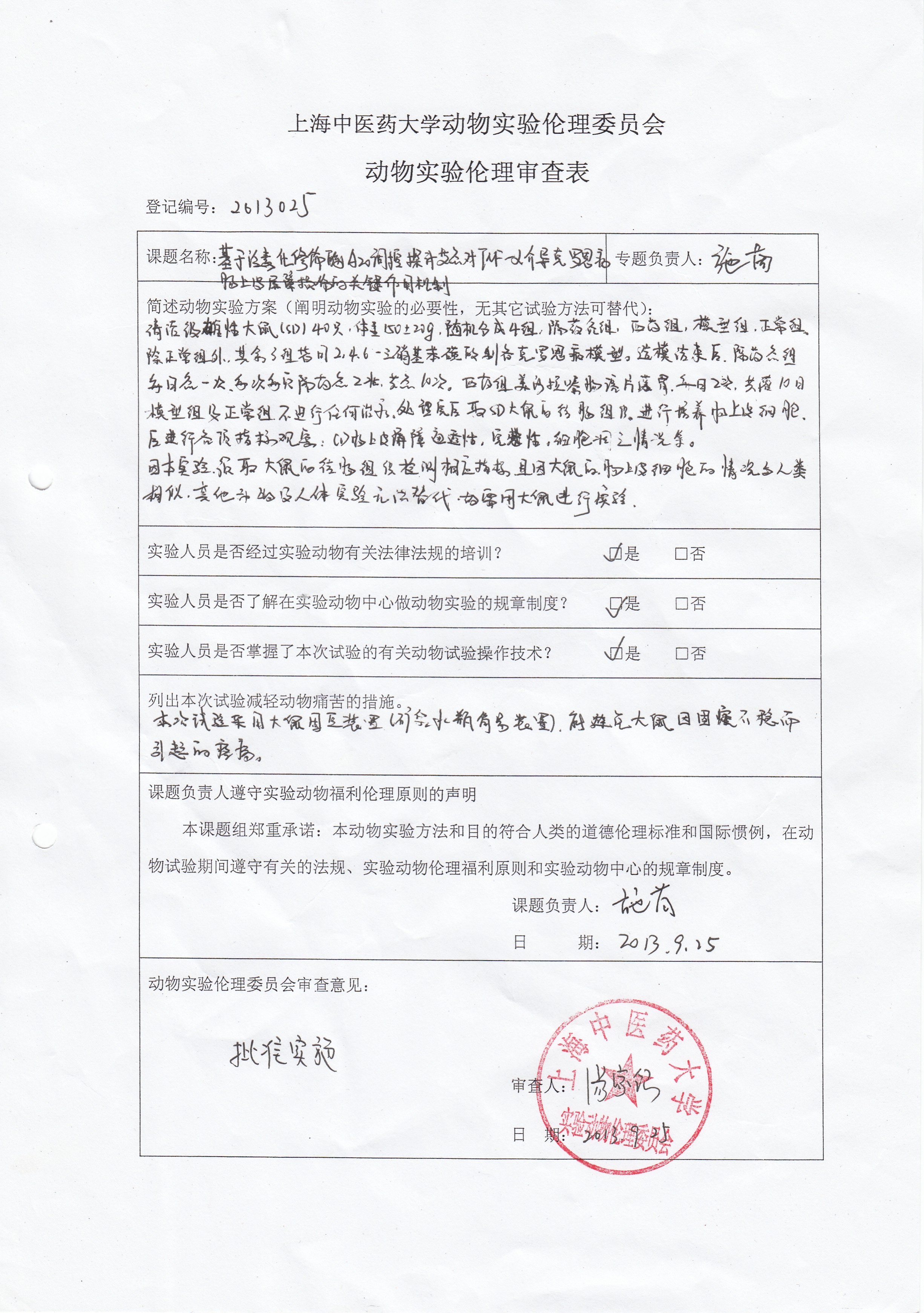

Supplement: Supplementary file 1 — 10.1186/s13020-016-0090-0 Ethical approval document in Chinese. [file 13020_2016_90_MOESM1_ESM.jpeg]

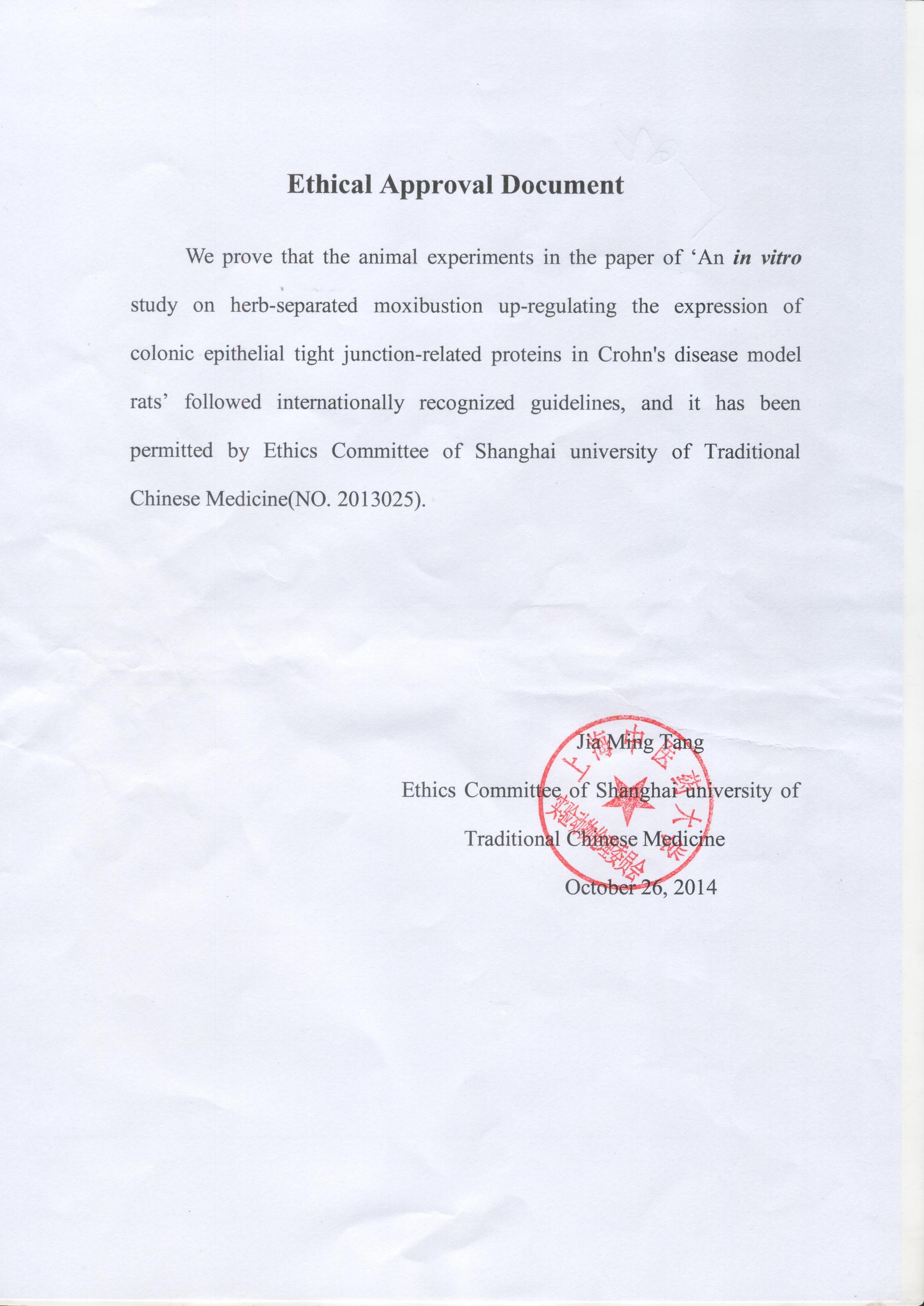

Supplement: Supplementary file 2 — 10.1186/s13020-016-0090-0 Ethical approval documents in English. [file 13020_2016_90_MOESM2_ESM.jpeg]
