# Supplementary material for: Herb-partitioned moxibustion upregulated the expression of colonic epithelial tight junction-related proteins in Crohn’s disease model rats
Source: Chin Med. 2016 Apr 26;11:20. doi: 10.1186/s13020-016-0090-0 (PMC4845475; doi:10.1186/s13020-016-0090-0)
Supplement: Supplementary file 3 — 10.1186/s13020-016-0090-0 The ARRIVE guidelines. [file 13020_2016_90_MOESM3_ESM.docx]

The ARRIVE Guidelines Checklist

ITEM RECOMMENDATION Section/Paragaph

| Title 1 Provide as accurate and concise a description of the content of the article as possible. | Herb-partitioned moxibustion  upregulated the expression of  colonic epithelial tightht junction-related proteins in in Crohn’s disease model rats |
| --- | --- |
| Abstract 2 Provide an accurate summary of the background, research objectives, including details of the species or strain of animal used, key methods, principal findings and conclusions of the study. | Abstract(including Background,Methods,Results and conclusion) |
| INTRODUCTION |  |
| Background 3 a. Include sufficient scientific background (including relevant references to previous work) to understand the motivation and context for the study, and explain the experimental approach and rationale.  b. Explain how and why the animal species and model being used can address the scientific objectives. | Background in main test |
| METHODS |  |
| Ethical statement 4 Indicate the nature of the ethical review permissions, relevant licences (e.g. Animal [Scientific Procedures] Act 1986), and national or institutional guidelines for the care and use of animals, that cover the research | The experiments were approved by Ethics Committee of Shanghai University of Traditional Chinese Medicine (No.2013025;Additional files 1 and 2) |
| Study design 5 For each experiment, give brief details of  t he study design including:  a. T he number of experimental and control groups.  b. Any steps taken to minimise the effects of subjective bias when allocating animals to treatment (e.g. randomisation procedure) and when assessing results (e.g. if done, describe who was blinded and when).  c. The experimental unit (e.g. a single animal, group or cage of animals).  A time-line diagram or flow chart can be useful to illustrate how complex study designs were carried out. | The section of “animals” in main test |
| Experimental 6 For each experiment and each experimental  procedures group, including controls, provide precise details of all procedures carried out. For example:  a. How (e.g. drug formulation and dose, site and route of administration, anaesthesia and analgesia used [including monitoring], surgical procedure, method of euthanasia). Provide details of any specialist  equipment used, including supplier(s).  b. When (e.g. time of day).  c. Where (e.g. home cage, laboratory, water maze).  d. Why (e.g. rationale for choice of specific anaesthetic, route of administration, drug dose used). | The section of “Treatment” in main test |
| Experimental 7 Provide details of the animals used,  Animals including species, strain, sex,  developmental stage (e.g. mean or  median age plus age range) and  weight (e.g. mean or  median weight plus weight range). | male Sprague-Dawley rats (150±10g), aged 6-8 weeks,  The section of “Animals” in main test |
| Sample size 8 Specify the total number of animals used in each experiment, and the number of animals in each experimental group. | the total number of animals is 48, the number of animals in each experimental group is 12 |
| Allocating animals to 9 a. Give full details of how animals were experimental groups allocated to experimental groups,including  Randommisation or matching if done.  b. Describe the order in which the animals in the different experimental groups were treated and assessed. | the rats were allocated into four groups, by a randomized block design. The details can be found in the section of “Animals”in main test.  The details about treatment can be found in the section of “Treatment”in main test |
| Experimental 10 Clearly define the primary and secondary  outcomes experimental outcomes assessed | 1、Assessment of macroscopic colonic damage  2、Establishment of an in vitro intestinal epithelial barrier model  3、Measurement of colonic TEER  4、Detection of occludin, claudin-1, and ZO-1 expression  5、FQ-PCRdetection of occludin, claudin-1, and ZO-1 mRNA expression |
| Statistical methods 11 Provide details of the statistical methods used for each analysis. | Statistical data were represented in graphs as means ± standard deviation (SD).  ifferences within experimental groups were compared by one-way analysis of variance (ANOVA). Values of *P* less than0.05 were considered statistically significant.LSD test was used for multiple comparision. |
| RRESULTS |  |
| Outcomes and estimation 12 Report the results for each analysis carried out, with a measure of precision (e.g. standard error or confidence interval ) | The details of results can be found  In the section of ‘Results’in main  test. |
| DISCUSSION |  |
| Interpretation/ 13 Interpret the results, taking into scientific implications account the study objectives and  hypotheses, current theory and other relevant studies in the literature. | Can be found in the section of “Discussion” |
| Funding 14 List all funding sources (including grant number) and the role of the funder(s) in the study. | The research was supported by a grant (No. 81273844) from the National Natural Science Foundation of China |
|  |  |
|  |  |
|  |  |
